# Champion Position Analysis in Short Track Speed Skating Competitions From 2007 to 2019

**DOI:** 10.3389/fpsyg.2021.760900

**Published:** 2021-12-07

**Authors:** Lixin Sun, Tianxiao Guo, Fei Liu, Kuan Tao

**Affiliations:** ^1^AI Sports Engineering Lab, School of Sports Engineering, Beijing Sport University, Beijing, China; ^2^School of Sports Science, Beijing Sport University, Beijing, China

**Keywords:** speed skating, short track, starting position, race analysis, performance

## Abstract

**Purpose:** Short track speed skating is a racing sport with racing tactics are equally crucial to speed and technical skating skills. Therefore, to investigate the relationship between starting and finishing positions for elite skaters and subsequently, explore pacing patterns for champions are necessary.

**Methods:** To investigate a pattern of effective tactical positioning strategy, Kendall’s tau-b correlations between starting and finishing position were calculated, with 500 m races having the most positive correlation (0.347, *P* < 0.05).

**Results:** Furthermore, starting position distributions of winners in each round, as well as the fluctuations in champion starting positions across rounds were analyzed. Our findings showed that skaters on the first track were inclined to win the rounds in 500, 1,000, and 1,500 m (28, 28, and 22%, respectively), and the differences between starting and finishing positions for champions were minimized in semi-finals. Meanwhile, the pacing patterns were gaining more fluctuations by the increase of race distances for champions, as the average standard deviation of lap rankings equaled 0.90, 1.15, and 2.21 for 500, 1,000, and 1,500 m races, respectively.

**Conclusion:** In conclusion, elite skaters should adopt flexible tactics at the lowest cost of energy consumption. The overall variability of lap ranking in long-distance races were distinctly higher than it in short distance.

## Introduction

Short track speed skating is a competitive way of ice speed skating. During races, competitors are pitched against each other in groups ranging from four to six and skate on an oval ice track with a length of 111.12 meters. The races are fast and furious with fastest speed reaching 91 kilometers per hour and skaters who continuously scramble for advantage often feel fatigue, frequently tackling with opponents to ground with them. Skaters are required to allocate the available energy that is known as pacing (Smits et al., 2014; Konings and Hettinga, 2018a). Many theories have suggested that the sensation of fatigue has a crucial impact on the decision-making process regarding sports performance (Schiphof-Godart and Hettinga, 2017), yet the detailed mechanism remains unclear. Elite short track speed skaters typically skate multiple races per day during tournaments, thus to perform optimally in head-to-head competitions, they need balance the optimal distribution of the available energy resource against possible tactic advantages or disadvantages (Hettinga et al., 2017).

In addition, short track speed skating is a racing game that tests the speed, technical skating ability and aggressiveness of its competitors. In contrast to conventional long-track speed skating, contestants race against each other instead of the clock. This means the final rankings are determined by the relative positions between skaters rather than the finishing time per short track speed skating player. To win the game, strength quality (Felser et al., 2016), push-off forces (Bullock et al., 2008), pacing behavior (Konings et al., 2016; Noorbergen et al., 2016; Menting et al., 2019) and race tactics (Bullock et al., 2008; Konings and Hettinga, 2018b) are important factors that matter to coaches and athletes. Therefore, the starting position analysis and the optimal pacing strategy of elite performance are crucial to acquire an effective tactical positioning strategy.

The lanes of the first qualifying round of skating are drawn by the Competitors Steward, a software that can assign random lane numbers to athletes, and for the subsequent rounds, the lane positions will be decided by the results from the preceding qualifying round according to the specific regulations of short track speed skating from ISU (International Skating Union) (International Skating Union, 2018). More specifically, the advanced athletes will first be regrouped into their ranks during the preceding round and then assigned lane positions, starting with the quickest time, from the inside to the outside of the track.

A study on the influence of starting position on finishing position in 500 m races showed a significant positive correlation between them especially in semi-finals and finals regardless of genders. This study was based on data from the five seasons between 1999–2000 and 2003–2004 of 500 m races at the ISU Short Track World Cups (Maw et al., 2006). Muehlbauer and Schindler (2011) extended the study of the relationship between the starting and finishing positions in all classifications based on the data from six competitions over three seasons from 2006–2007 to 2008–2009 and found that the correlation between the two positions decreased with race distance, which was highest and positive in the 500 m races, lowest in the 1,500 m races and negative in the 3,000 m races and showed no gender difference. A more recent study in 2015 by Haug et al. (2015) also focused on World Cup 500 m races, but more concerned with the association between start performance evaluated by first-corner position entering (RPEFC) ratings and race results from elite athletes. Since videos had to be used, data acquisition by the RPEFC was limited due to labor-intensive workload.

As a result, finishing times are irrelevant in short track speed skating as long as skaters finish ahead of opponents. The current studies explored the differences in variability between and within races, and demonstrated how analyses of such variability could provide insights into tactical pacing decisions (Konings and Hettinga, 2018a,b). More specifically, the preceding high-intensity races efforts in a competitive weekend affected pacing and performance of elite skaters (Konings and Hettinga, 2018b). For instance, adolescent skaters were assessed the quality of overtaking maneuvers to improve tactics in training process (Martynenko and Oreshkina, 2020), while coaches should inform young athletes regarding the velocity distribution between race types in order to reserve energy and achieve a higher velocity in the final section (Menting et al., 2020).

Although position analysis and pacing strategy have demonstrated prominent roles in short track speed skating races, current researches on this topic are inadequate with limited data, and yet no literature, to the best of our knowledge, is dedicated to the exploration of pacing patterns for champions. This study will first verify the previous findings with a new larger dataset, and then focus on the winners in each round to investigate the distribution of their starting positions. The variations of champion starting positions during the rounds are also explored to examine positioning strategies of these medalists. Finally, the pacing patterns for champions in 500, 1,000, and 1,500 m events are rigorously analyzed.

## Materials and Methods

### Data Acquisition

This paper deeply investigated the relationship between the positions and rankings from 121 competitions (World Cup Series / Championships / Olympic Games) from 2007/2008 to 2019/2020 season. All data were created and assessed the validity and reliability from the official website of International Skating Union (ISU), under the consent of the terms and conditions of the data source. The original dataset comprised of 183,158 items of match information (714 female and 904 male athletes), with each piece representing split time of each athlete, name of competitions, rounds and laps numbers. After data cleaning, which attempted to discard data when penalties or disqualifications occurred, 67,258 pieces of data altogether containing the temporal information about 500, 1,000, and 1,500 m competitions in the preliminary, heat, quarter-final, semi-final and final rounds were thus eliminated. Hence, filtered dataset consisted of 115,900 pieces of data which were labeled by sorts of attributes, such as race distance, competition, gender, name, country and so forth, would be conducted via multi-dimensional analysis. For further analysis of race behaviors for champions, 173 female and 247 male champions were announced out of 4,313 female and 5,212 male individual races (preliminaries, heats, quarter-finals, semi-finals, and final in 500, 1,000, 1,500, and 3,000 m classifications).

### Statistical Analysis

Starting positions were marked as 1 to 4 (500 and 1,000 m), and 1 to 6 (1,500 m) that represent inner to outer lanes. The Kendall’s tau-b correlation was analyzed to evaluate the correlation between starting and finishing positions of the race. The correlation coefficient interpretations vary with magnitudes of values lower than 0.2 or 0.2 to 0.5 or greater than 0.5, suggesting low, moderate and strong correlations, respectively. And the significance level was set as P < 0.05. Correlation coefficient analysis was conducted via the SPSS (version 16.0). Moreover, we sketched the starting position distributions for round winners and obtained the dynamical evolution between starting and finishing positions in different rounds for champions in order to realize the most optimal match strategy. We also calculated the mean value and standard deviation of rankings per laps for champions in all rounds to obtain pacing patterns and individual skating styles for elite skaters.

## Results

### Correlations Between Starting and Finishing Position

The correlations between starting and finishing position by different race distances and rounds were shown in Table 1. These correlations were all significant, with the largest value appearing in the event of 500 m (0.347, *P* < 0.05) and progressively decreasing by 1,000 m (0.194, *P* < 0.05) and 1,500 m (0.133, *P* < 0.05). An exception was 3,000 m, because the correlation coefficient evidently exceeded 1,000 and 1,500 m (0.286, *P* < 0.05). To figure out the abnormal phenomenon of 3,000 m, the correlations of 3,000 m Super-final and 3,000 m Relay were further calculated, respectively. 3,000 m Super-final race displayed the lowest correlation coefficient (−0.006, *P* < 0.05) in all the race distances while the 3,000 m Relay (0.281, *P* < 0.05) supplied the highest value.

**TABLE 1:** Pairwise correlation of rounds in different events.

	500 m	1,000 m	1,500 m	3,000 m
Preliminaries	0.127**	0.080**	0.063**	–
Heats	0.474**	0.214**	0.090**	0.099**
Quarter-finals	0.500**	0.265**	0.154**	0.272**
Semi-finals	0.465**	0.219**	0.162**	0.397**
Final	0.429**	0.247**	0.168**	0.351**
All	0.347**	0.194**	0.133**	0.286**

** Means all the values are statistically significant.

Furthermore, there were various trends in correlation coefficients across rounds in different race distances. Like 500 m of the column (Table 1), the correlation coefficients of preliminaries were noticeably lower than others (0.127, *P* < 0.05) and the similar tendency was found in 1,000 m race although all the rounds were positively correlated. On the contrary, in 1,500 and 3,000 m race, correlation coefficients of Heats were also far lower than the final rounds. There was no specific linear relation about the correlation between starting and finishing position among quarter-final, semi-final and final rounds.

This research mainly concentrated on the individual races, so that only 500, 1,000, and 1,500 m races were further investigated. Gender in this research was not considered since no conclusions on sexually significant differences had been derived from prior studies.

### Starting Position Distribution for Round Winners

**FIGURE 1:**
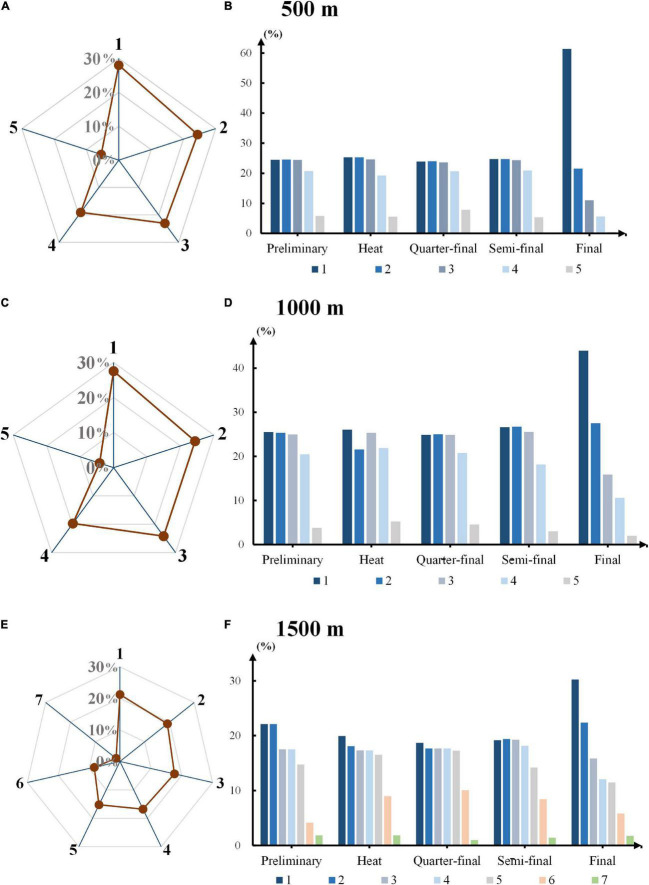
Starting position distribution for round winners. **(A,C,E)** The overall starting position distribution of the winners in different race distances in 500, 1000, and 1500 m competitions respectively. The axis labels on radar charts represent starting lanes. **(B,D,F)** Separate starting position distributions of each round ranging from preliminary to final in 500, 1000, and 1500 m competition.

Figure 1 showed the overall starting position distribution of the winners in different race distances in Figures 1A,C,E, and separate distribution of each round in Figures 1B,D,F correspondingly. Starting position 1 had the highest probabilities to be round winners (28%, 28%, 22%) on all three race distances, the close margins suggested that advantages of starting lanes mattered in some extent. Athletes with first position were obviously advantageous to win the final rounds (61%, 44%, 30%) when we observed the histograms of each race distance. The winners were, however, largely split amongst the top 3 starting lanes in preliminary, heat, quarter-final and semi-final rounds. Skaters in last few lanes always provided fewer victors in all kinds of race and rounds.

### Dynamical Evolution of Starting and Finishing Position for Champions

**FIGURE 2:**
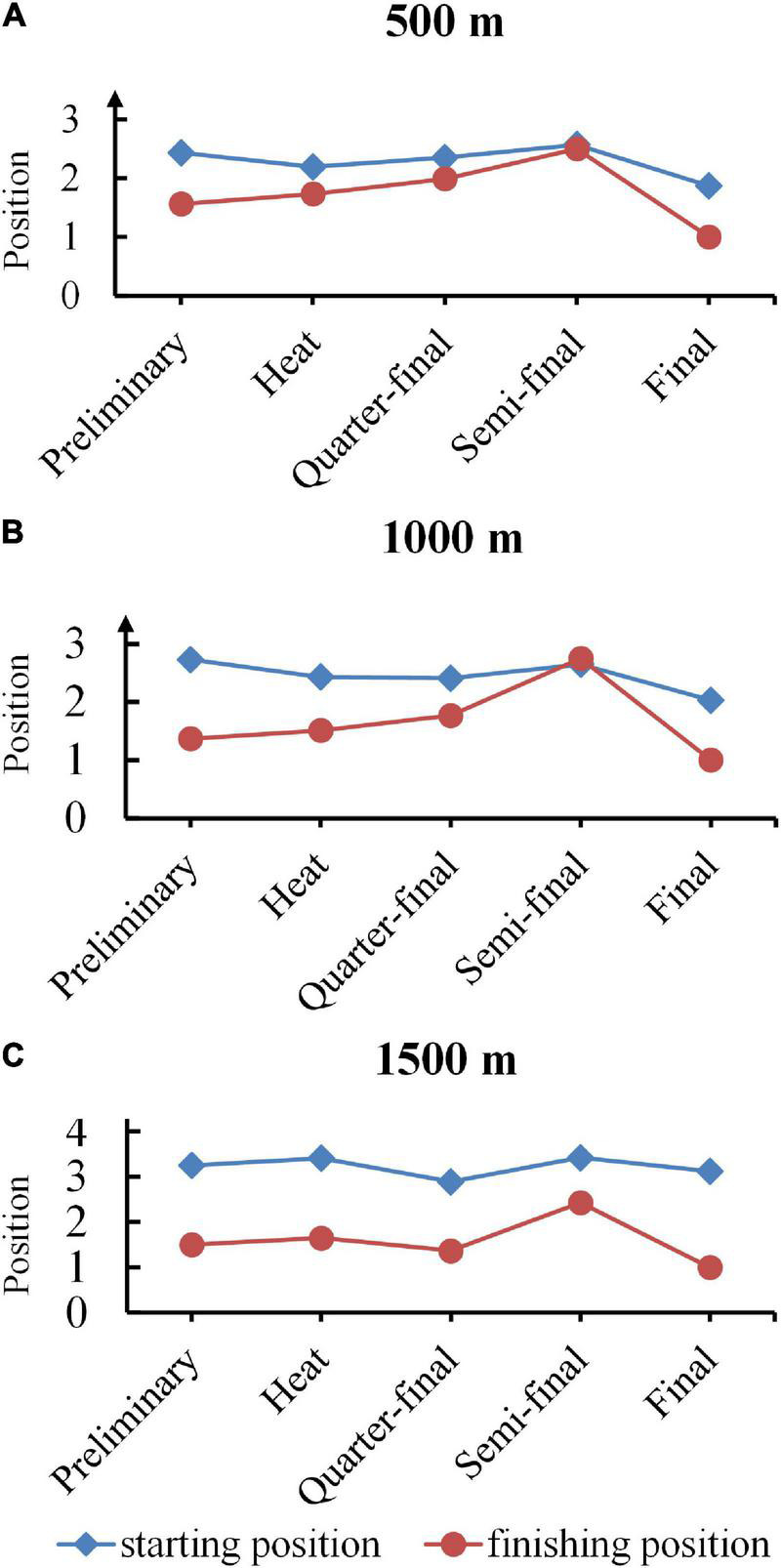
Dynamical evolution of starting and finishing position for champions. **(A–C)** Profiles of starting and finishing position for 500, 1000, and 1500 m champions. The blue broken line with a square mark represented starting positions, while the red one with a circle represented finishing positions.

Figure 2 depicted the dynamical evolutions of mean starting and finishing positions, which were calculated in order to investigate the performances from hundreds of champions in every qualifying round. Dynamical evolution was defined as the variation of positions per skater with different rounds in short track speed games, which revealed the various game tactics that skaters adopted. The blue broken line with a square mark represented starting positions, while the red one with a circle represented finishing positions. The 500 and 1,000 m graphs revealed a similar pattern, where mean starting position and finishing position overlapped on the semi-final. The final position graph of three race distances shared a common bell-shaped trend with respect to the peak of semi-final, implying that the ranking output of semi-final was typically lower than other qualifying rounds. Above all, mean starting positions in all race distances and qualifying rounds were less than 3 (out of 4), mean finishing positions of 500 and 1,000 m were less than 3 (out of 4) and 1,500 m less than 4 (out of 6).

### Pacing Patterns in Each Round for Champions

**FIGURE 3:**
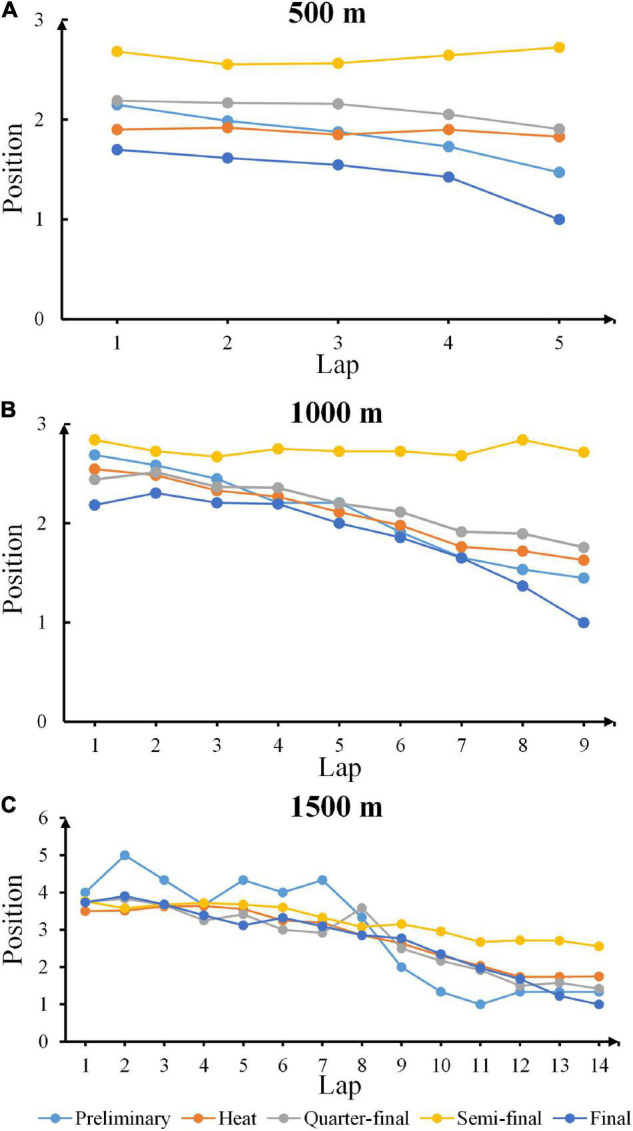
Pacing patterns in each round for champions. **(A–C)** The pacing patterns for champions during each round in 500, 1,000, and 1,500 m short track speed skating competitions. The light blue, orange, gray, yellow, and dark blue stand for preliminary, heat, quarterfinal, semi-final, and final rounds, respectively.

Figure 3 illustrated the pacing patterns (defined as the clusters and similarities per skater with laps to go in each game) for champions during each round in 500, 1,000, and 1,500 m short track speed skating competitions. The light blue, orange, gray, yellow and dark blue stand for preliminary, heat, quarter-final, semi-final and final rounds, respectively. The pacing patterns were gaining more fluctuations by the increase of race distances, with the maximum margin of position per lap in all rounds was smaller than 0.7 in 500 m competitions, while this value reached 1.2 in 1,000 m and exceeded 2.7 in 1,500 m. Meanwhile, the average standard deviation of position per lap equaled 0.90, 1.15, and 2.21 for 500 m, 1,000 and 1,500 m races, respectively, indicating that in the event of 1,500 m race, champions from varied competitions might adopt completely different pacing patterns.

The mean values and standard deviations of lap rankings for champions in 500 m, 1,000 and 1,500 m were computed (Tables 2–4), respectively, indicating that the overall variability of lap ranking reduced with the increasing laps, and standard deviations for longer distance races were distinctly higher than for short distance like 500 m. These findings revealed that long-distance race champions started from widely dispersed places but concluded with similar patterns. And large amount of laps naturally provided greater space for allocating energy and adjusting strategies.

**TABLE 2:** Mean value and standard deviation of lap rankings for 500 m champions.

500 m					
Lap	Final	Semi-final	Quarter-final	Heat	Preliminary
1	1.7 ± 0.9	2.7 ± 1.3	2.2 ± 1.1	1.9 ± 1.0	2.1 ± 1.1
2	1.6 ± 0.7	2.6 ± 1.4	2.2 ± 1.1	1.9 ± 0.9	2.0 ± 1.0
3	1.5 ± 0.6	2.6 ± 1.2	2.2 ± 1.0	1.9 ± 0.9	1.9 ± 0.9
4	1.4+0.5	2.6 ± 1.2	2.1 ± 0.9	1.9 ± 0.9	1.7 ± 0.7
5	1.0 ± 0	2.7 ± 1.1	1.9 ± 0.7	1.8 ± 0.7	1.5 ± 0.6

**TABLE 3:** Mean value and standard deviation of lap rankings for 1,000 m champions.

1,000 m					
Lap	Final	Semi-final	Quarter-final	Heat	Preliminary
1	2.2 ± 1.0	2.8 ± 1.6	2.4 ± 1.5	2.5 ± 1.4	2.7 ± 1.5
2	2.3 ± 0.9	2.7 ± 1.7	2.5 ± 1.3	2.5 ± 1.3	2.6 ± 1.3
3	2.2 ± 1.0	2.7 ± 1.7	2.4 ± 1.2	2.3 ± 1.4	2.4 ± 1.3
4	2.2 ± 1.1	2.8 ± 1.7	2.4 ± 1.4	2.3 ± 1.3	2.2 ± 1.3
5	2.0 ± 1.0	2.7 ± 1.6	2.2 ± 1.5	2.1 ± 1.2	2.2 ± 1.3
6	1.9 ± 0.9	2.7 ± 1.7	2.1 ± 1.2	2.0 ± 1.1	1.9 ± 0.9
7	1.7 ± 0.7	2.7 ± 1.6	1.9 ± 1.1	1.8 ± 0.9	1.7 ± 0.7
8	1.4 ± 0.4	2.8 ± 1.5	1.9 ± 1.1	1.7 ± 0.8	1.5 ± 0.6
9	1.0 ± 0.0	2.7 ± 1.2	1.8 ± 0.6	1.6 ± 0.7	1.4 ± 0.5

**TABLE 4:** Mean value and standard deviation of lap rankings for 1,500 m champions.

1,500 m					
Lap	Final	Semi-final	Quarter-final	Heat	Preliminary
1	3.7 ± 3.1	3.8 ± 3.6	3.8 ± 3.2	3.5 ± 3.1	4.0 ± 2.0
2	3.9 ± 3.3	3.6 ± 3.4	3.8 ± 3.5	3.5 ± 3.3	5.0 ± 0
3	3.7 ± 3.7	3.7 ± 3.5	3.7 ± 4.2	3.6 ± 3.0	4.3 ± 0.9
4	3.4 ± 3.5	3.7 ± 3.6	3.3 ± 4.2	3.6 ± 3.1	3.7 ± 3.6
5	3.1 ± 3.2	3.7 ± 3.3	3.4 ± 4.2	3.6 ± 2.9	4.3 ± 0.9
6	3.3 ± 2.6	3.6 ± 3.2	3.0 ± 4.2	3.3 ± 3.2	4.0 ± 0.7
7	3.1 ± 2.8	3.3 ± 3.1	2.9 ± 3.6	3.2 ± 3.3	4.3 ± 0.2
8	2.9 ± 3.1	3.1 ± 3.1	3.6 ± 3.6	2.9 ± 2.9	3.3 ± 0.9
9	2.8 ± 2.5	3.2 ± 3.2	2.5 ± 1.3	2.6 ± 2.4	2.0 ± 2.0
10	2.3 ± 2.2	3.0 ± 2.8	2.2 ± 1.0	2.3 ± 1.7	1.3 ± 0.2
11	2.0 ± 1.3	2.7 ± 2.5	1.9 ± 1.1	2.0 ± 1.3	1.0 ± 0
12	1.7 ± 1.0	2.7 ± 2.4	1.5 ± 0.4	1.7 ± 0.9	1.3 ± 0.2
13	1.2 ± 0.5	2.7 ± 2.3	1.6 ± 0.6	1.7 ± 0.9	1.3 ± 0.2
14	1.0 ± 0	2.6 ± 1.7	1.4 ± 0.4	1.8 ± 0.7	1.3 ± 0.2

## Discussion

The purposes of the present study are twofold. Firstly, investigate the relationship between starting and finishing positions for elite skaters in short track speed skating races. Secondly, explore pacing patterns for champions. Consequently, four statistical experiments were progressively conducted. (i) We calculated the correlations between starting and finishing position on the basis of rounds in all competitions to investigate the overall impacts of starting lanes on final ranking. (ii) The starting position distribution of winners was examined accordingly in each round based on the first part. (iii) Dynamical evolution of starting and finishing positions were analyzed for champions. These sequential characteristics revealed the strategic patterns of elite short track speed skating athletes. (iv) Pacing patterns for champions in varied competitions were studied as we examined the position per lap along with standard deviation and the maximal margin.

The positive correlations are seen in 500 to 3,000 m events, which are consistent with findings in Maw et al. (2006) and Muehlbauer and Schindler (2011). Interesting to note that the magnitude of correlation reduces as race distances increase, which suggests that pacing strategies vary as race distances increase. One possible explanation accounts for such phenomenon is that longer distance is accompanied with more expense of energy. Maintaining a leading position in a 500 m race, for example, consumes less energy than in a 1,000 or 1,500 m race (Di Prampero et al., 1976). In addition, a negative correlation was obtained in the 3,000 m Super-finals. In terms of energy, drafting is more efficient and less exhausting than leading in long races (Kyle, 1979). Additionally, the starting and finishing positions are closest in semi-finals for champions since short track speed skating contests are elimination events, which means that performance gaps between skaters shrink from preliminary to final. Elite skaters might adopt a strategy to conserve energy during the qualifying rounds before to the semi-finals, however, they must maintain excellent performance to advance to the final round. Besides, skaters on the first track are inclined to win the rounds in 500, 1,000, and 1,500 m (28, 28, and 22%, respectively) after computing the mean positions for winners from preliminary to final qualifying round. Although starting positions are picked at random in the preliminary, faster skaters acquire inner starting positions relative to slower skaters in the subsequent rounds, making it more difficult for opponents to overtake.

To seek for the theoretically optimal distribution of energy and improve the tactical considerations have already gained much attention in cross-country running (Hanley, 2014), rowing (Edwards et al., 2016), and track cycling (Moffatt et al., 2014). However, for short track speed skating, the impact of preceding high intensity efforts on pacing and performance is significant (Konings and Hettinga, 2018b). Interesting to note that although champions from varied competitions adopt different strategies to accelerate, semi-final races exhibit the least fluctuations in positions (mean position ranking equals 2.63, 2.74, and 2.98 in 500, 1,000, and 1,500 m correspondingly). One possible reason is that no elimination happens in this round therefore skaters conserve energy in order to outperform opponents in final rounds. The findings in Figure 3 further confirm it because obvious accelerations are obtained in final rounds whatever 500, 1,000, and 1,500 m races. Another interesting behavior is that pacing patterns for champions drastically vary in 1,500 m race. The potential explanation for this phenomenon lies in the fact that skaters undergo more laps compared to the event of 500 and 1,000 m, which yields more complicated strategical overtakes. At the meantime, the total players per round outnumber than those in 500 and 1,000 m races which result in the fluctuation of positions per lap for champions in each round as being an all-time leader undertakes more energy consumption, and, to some extent, at a higher risk of being overtaken.

The use of artificial intelligence technologies to visualize and analyze data gains favor in modern sports science (Guo et al., 2020), due to its outstanding abilities for generalization and efficiency in investigation of the non-linear latent interactions between variables. We accept that some limitations exist in the present study, such as the involvement of just starting and finishing positions instead of incorporating more detailed information gathered from race profiles (i.e., split time). And any disqualifications or penalties are ruled out during the data cleaning process, which, however, deserves a thorough analysis in the future work since these unintentional occurrences have an influence on the modifications of racing strategy for skaters.

### Practical Applications

Our findings indicate that elite skaters should adopt a strategy to conserve energy during the qualifying rounds before coming to the semi-finals, however, they should maintain excellent performance to advance to the final round. Meanwhile, round winners were largely split amongst the top 3 starting lanes in preliminary, heat, quarter-final and semi-final races in 500, 1,000, and 1,500 m competitions. Although long-distance race champions started from widely dispersed places but finished with similar pacing patterns, the overall variability of lap ranking in 1,500 m races were distinctly higher than it in short distance (500 m races).

## Data Availability Statement

The raw data supporting the conclusions of this article will be made available by the authors, without undue reservation.

## Author Contributions

LS and KT designed the study. KT supervised the study. TG and FL performed the experiments. LS, TG, and KT wrote the manuscript. All authors contributed to the article and approved the submitted version.

## Conflict of Interest

The authors declare that the research was conducted in the absence of any commercial or financial relationships that could be construed as a potential conflict of interest.

## Publisher’s Note

All claims expressed in this article are solely those of the authors and do not necessarily represent those of their affiliated organizations, or those of the publisher, the editors and the reviewers. Any product that may be evaluated in this article, or claim that may be made by its manufacturer, is not guaranteed or endorsed by the publisher.
